# Alveolar ridge preservation using an open membrane approach for sockets with bone deficiency: A randomized controlled clinical trial

**DOI:** 10.1111/cid.12668

**Published:** 2018-11-05

**Authors:** Dong‐Joo Sun, Hyun‐Chang Lim, Dong‐Woon Lee

**Affiliations:** ^1^ Department of Periodontology Veterans Health Service Medical Center Seoul Republic of Korea; ^2^ Department of Periodontology School of Dentistry, Kyung Hee University Seoul Republic of Korea

**Keywords:** alveolar ridge preservation, bone resorption, dense polytetrafluoroethylene membrane, freeze‐dried allogenic bone

## Abstract

**Background:**

Various approaches are used for alveolar ridge preservation (ARP); however, there is no standard method or material.

**Purpose:**

To investigate the effect of ARP with a dense polytetrafluoroethylene (d‐PTFE) membrane and freeze‐dried irradiated allogenic bone for sockets with bone deficiency.

**Materials and Methods:**

Thirty‐four patients (with sockets exhibiting ≥3 mm hard tissue loss in ≥1 walls) were randomized to undergo natural socket healing (control) or ARP with a d‐PTFE membrane and freeze‐dried irradiated allogenic bone (test group). After 4 months, horizontal and vertical ridge changes were measured using cone beam computed tomography.

**Results:**

Ridge width at l mm below the ridge crest demonstrated significantly less change in the test group (median =2.3; Q1 = 0.6; Q3 = 4.3 mm) than in the control group (median =3.9; Q1 =2.6; Q3 = 7.8 mm; *P* = .021). There was no significant difference between the two groups in horizontal ridge changes at 3 and 5 mm below the crest or vertical changes (*P >* .05). Requirement for bone augmentation at implant placement was significantly reduced in the test group compared to the control group (*P* < .001).

**Conclusion:**

ARP with a d‐PTFE membrane and freeze‐dried irradiated allogenic bone substitute reduced horizontal bone resorption in sockets with bone deficiency.

## 1 INTRODUCTION

Natural healing following tooth extraction is always accompanied by ridge shrinkage. The shrinkage occurs mostly during the early healing period (ie, within 3 months) and continues up to 12 months,^1^ which can compromise implant placement and esthetic restoration.^2^ Therefore, treatment planning should include consideration of maintaining alveolar ridge dimension, which is called alveolar ridge preservation (ARP).^3^ Several systematic reviews and meta‐analyses have demonstrated that ARP significantly reduces ridge shrinkage compared to natural socket healing.^3^, ^4^, ^5^, ^6^, ^7^ However, there is substantial heterogeneity in the surgical methods and materials used for ARP.^7^

Some ARP studies have attempted primary wound closure based on the similar concept of guided bone regeneration.^8^, ^9^ Membrane exposure, especially when using an expanded polytetrafluoroethylene (e‐PTFE) membrane, is considered to be detrimental because it increases the risk of infection and disturbs bone formation^10^; however, in the context of ARP, iatrogenic or intentional exposure of a collagen membrane and a dense polytetrafluoroethylene (d‐PTFE) membrane was acceptable and did not interfere with bone formation.^11^, ^12^, ^13^, ^14^ There are some differences between a collagen membrane and d‐PTFE membrane with regard to the healing process; collagen membrane resorbs naturally into the host tissue^15^ and permits blood vessel penetration,^16^ while d‐PTFE does not allow blood vessels or other tissues to pass through the membrane.^17^

ARP procedures can be successfully performed using various bone substitute materials, such as autografts, xenografts, allografts, and alloplasts.^18^ The choice of bone substitute material may depend on the preference of the clinician, funding, or cultural background. Among the bone substitute materials, freeze‐dried bone allograft has been one of the frequently used biomaterials for ARP.^4^ Evidence indicates that freeze‐dried bone allograft provides a scaffold for osteogenic cell migration as well as space maintenance.^19^, ^20^ The effect of this bone substitute on ARP has been demonstrated clinically, radiographically, and histologically.^11^, ^21^, ^22^ A recent Bayesian network meta‐analysis even demonstrated that freeze‐dried bone graft with a membrane shows superior effectiveness in the reduction of bone height remodeling compared with other modalities.^4^

Currently, most studies on ARP have been conducted on sockets with minimal bone deficiency.^23^ However, many teeth requiring extraction in an everyday clinical setting demonstrate more severe bone deficiency in the alveolus than that in previous clinical trials. Accordingly, the aims of the present study were to investigate (1) radiographic ridge changes following ARP with a d‐PTFE membrane and freeze‐dried irradiated allogenic bone for sockets with bone deficiency and (2) implant‐related outcomes.

## 2 MATERIALS AND METHODS

### 2.1 Study design

The present study was a prospective, randomized, parallel‐arm, controlled clinical trial. The research protocol was approved by the Institutional Review Board of VHS Medical Center (BOHUN 2016‐05‐002) and all patients provided written informed consent. The study was conducted in accordance with the Helsinki Declaration of 1975 and its later revisions. Also, the study was registered with the Republic of Korea Clinical Trials Registry (Identifier Number: KCT0002872).

### 2.2 Study population

Patients were enrolled between May 31, 2016 and November 16, 2016. All participants were informed about the details and purpose of the study, underwent an examination of the potentially eligible teeth, and provided written informed consent prior to study participation. All patients received proper periodontal treatment prior to commencing the study procedures when necessary. The inclusion criteria were as follows: (1) age ≥ 19 years, (2) types 3 or 4 extraction socket morphology (≥3 mm of hard tissue loss in 1 or more socket walls) according to the extraction socket classification,^24^ (3) no systemic disease contraindicating surgical procedures or compromising wound healing, and (4) healthy or stable periodontal status (bleeding on probing and plaque index <25%). The exclusion criteria were as follows: (1) current smoker (≥10 cigarettes per day), (2) pregnancy or lactation, (3) uncontrolled or untreated periodontal disease, and (4) inability to understand the trial purpose and provide informed consent.

### 2.3 Study groups

In the control group, the socket was allowed to heal naturally. In the test group, the socket was filled with freeze‐dried irradiated allogenic bone (ICB Cortical, Rocky Mountain Tissue Bank, Aurora, Colorado) and covered with a d‐PTFE membrane (OpenTex, Purgo, Seoul, Korea). No primary wound closure was attempted.

### 2.4 Randomization and allocation concealment

Each patient was randomly allocated to the control group or test group using computer‐generated randomization. Group allocations were concealed in opaque envelopes by an independent investigator. Envelopes were opened after tooth extraction and degranulation to identify treatment group assignments.

### 2.5 Outcome measures

#### 2.5.1 Primary outcome

Changes in the horizontal ridge width at a levels of 1, 3, and 5 mm below the ridge crest (HW1, HW3, and HW5),^25^, ^26^ assessed using cone beam computed tomography (CBCT).

#### 2.5.2 Secondary outcomes

Changes in the vertical ridge height at the buccal, mid, and lingual crests (VHB, VHM, and VHL, respectively),^27^ assessed using CBCT.

The need for an additional bone augmentation at the time of implant placement.

### 2.6 Surgical procedures

**Figure 1:**
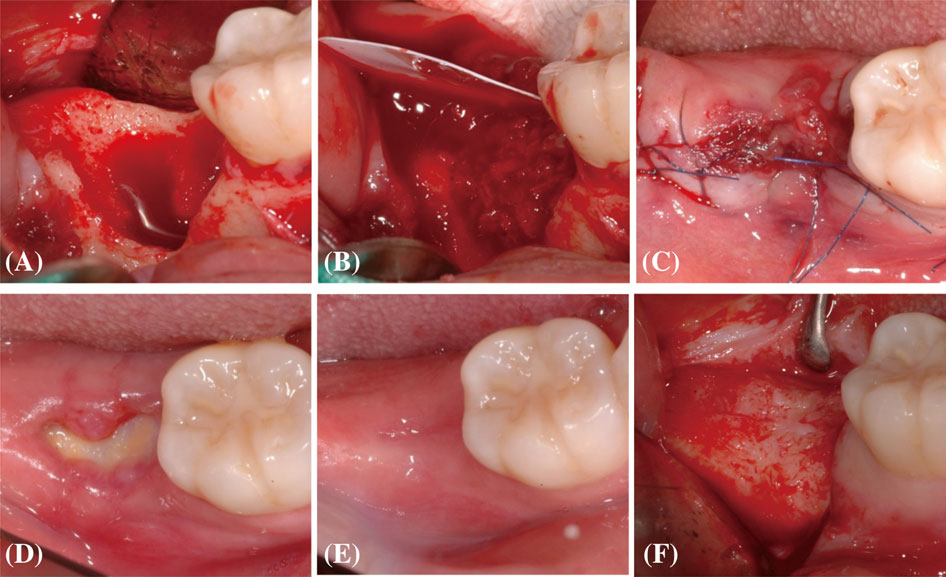
Representative clinical photographs of the test group after extraction (A), during the grafting of bone substitute material and d‐PTFE membrane placement (B), at flap approximation (C), 1 month after alveolar ridge preservation (D), 4 months after alveolar ridge preservation (E), and at the time of implant placement (F)

**Figure 2:**
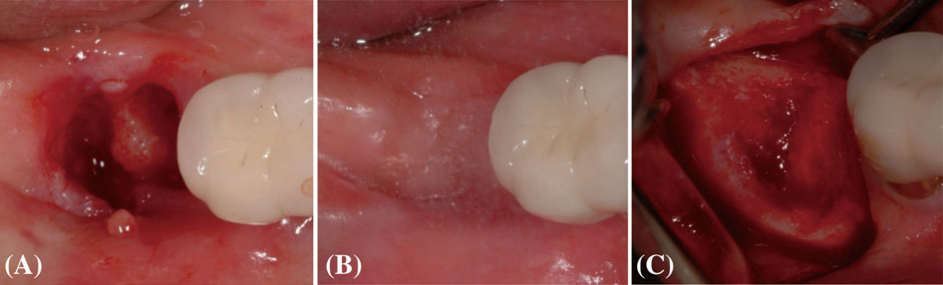
Representative clinical photographs of the control group after extraction (A), after 4 months of healing (B), and at the time of implant placement (C)

Sulcular incisions were performed around the recipient and adjacent teeth under local anesthesia and the periodontal flap was elevated. Upon identification of a bone defect with heavy tissue granulation, a vertical incision was made to visualize the surgical site and gentle extraction was performed with meticulous debridement. Sockets assigned to the test group were filled with freeze‐dried irradiated allogenic bone substitute particles and covered with a d‐PTFE membrane. The membrane covered at least 2 to 3 mm beyond the defect margin. The flaps were sutured using interrupted and horizontal mattress suturing. Primary wound closure was not attempted (Figures 1 and 2). CBCT (voxel size: 0.40 mm, exposure time: 8.9 seconds, 120 kVP, 18.54 mAs) using a KaVo 3D eXam instrument (Imaging Sciences International LLC, 1910 North Penn Road Hatfield, Pennsylvania) was performed immediately after surgery. Patients were instructed to rinse twice daily with a chlorhexidine gluconate solution (Hexamedine; Bukwang, Seoul, Korea) and prescribed analgesics and antibiotics for 3 to 5 days. All patients were followed‐up 7 to 10 days after the procedure for the removal of suture materials. The d‐PTFE membrane was removed without anesthesia 1 month after the surgery.

### 2.7 Follow‐up and implant placement

Patients were followed‐up regularly after ARP. Four months after the ARP or extraction procedure, another CBCT scan was performed and implants were placed. Bone augmentation was performed in the test and control groups when indicated. Bone core biopsy was performed in the test group when possible.

### 2.8 CBCT analysis

Radiographic measurements were performed by D.J.S. under supervision of the senior author (D.W.L.). Ten random cases were selected and measured twice to confirm reproducibility. The intra‐class correlation coefficient was in the range of 0.695 to 0.919 (*P* < .05).

Two CBCT scans (immediately after ARP or extraction and at 4 months postprocedure) were superimposed using stable references (eg, the cranial base or palatal vault for the maxilla and the inferior border for the mandible) and further manual correction was performed for best‐matched cuts.^25^, ^26^ A vertical reference line was drawn along the center of the socket considering the long axis of the extracted tooth and adjacent tooth. Then, two lines parallel to the vertical reference line were made passing through the buccal and lingual crests. Horizontal reference lines were drawn perpendicular to the vertical line at 1, 3, and 5 mm below the alveolar crest. These lines were used to measure horizontal changes at HW1, HW3, and HW5, and vertical changes, that is, VHB, VHM, and VHL.

### 2.9 Histological processing and histomorphometric analysis

The harvested bone cores were fixed in 10% buffered neutral formalin (Sigma Aldrich, St. Louis, Missouri) for 14 days. Then, bone cores were decalcified in 5% formic acid and embedded in paraffin. Serial perpendicular sections (5‐μm thickness) were cut along the center of each specimen, and the central‐most sections were stained with hematoxylin and eosin as well as Masson's trichrome. A histomorphometric analysis was performed using image analysis software (Photoshop CS6, Adobe, California). The percentages of newly formed bone (NB), residual bone substitute material (RM), and soft tissue and background (SB) were measured.

### 2.10 Statistics

The required sample size was calculated using G*power software (ver. 3.1, Faul, F., Erdfelder, E., Lang, A.‐G., & Buchner, Germany). Previous studies by Engler‐Hamm and colleagues and Schropp and colleagues were used to estimate the effects of ARP and dimensional changes after tooth extraction,^1^, ^13^ assuming an effect size of 1.12. The sample size required for the present trial was at least 15 patients (one tooth per patient) per group to obtain 80% power with an alpha level of 0.05.

Data are presented as mean, standard deviation, median and quartiles. Shapiro‐Wilk tests were used to verify the normal distribution of variables. Mann‐Whitney *U* tests were used to compare changes in ridge width and height between groups. Additionally, non‐molar and molar sites were pooled separately, and descriptive statistics were used. Pearson chi‐square tests were used to examine between‐group differences in the need for bone augmentation. The threshold for statistical significance was set at *P* < .05.

## 3 RESULTS

**Figure 3:**
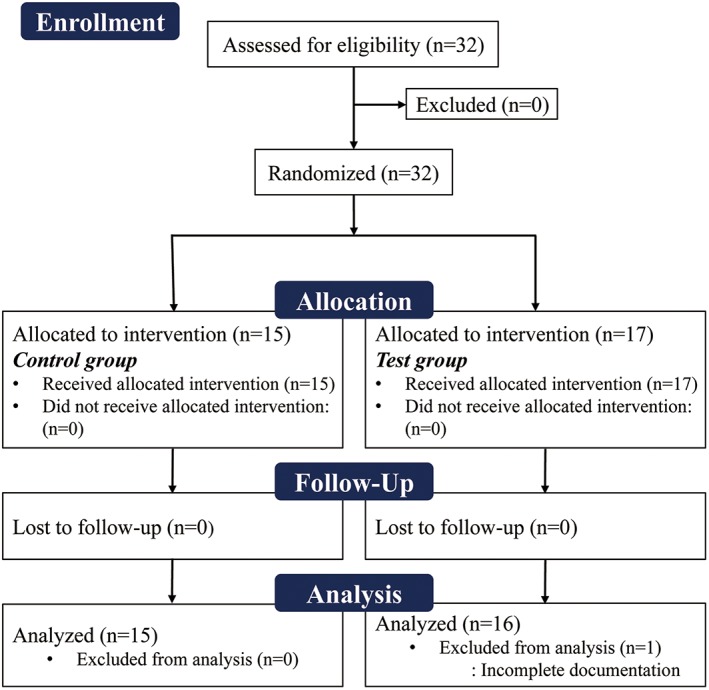
Flowchart of patient enrollment, randomization, and allocation

Thirty‐two patients (38 extraction sockets) were enrolled in the present study. For patients requiring more than one tooth extraction, a single socket was randomly selected. One patient in the test group dropped out due to incomplete documentation. Therefore, 31 patients (control group: 15, test group: 16) with a mean age of 67.8 ± 7.3 years (range, 42‐80) completed the study and were included in the analysis (Figure 3). Nineteen sockets were in the maxilla and 12 sockets were in the mandible. Sixteen sockets were nonmolar sites and 15 were molar sites. Tooth extraction was mainly necessitated by periodontitis (*n* = 19) or tooth fracture (*n* = 12), but all teeth with fracture also exhibited alveolar bone loss. All patients received the intended surgical intervention as per group allocation (Table 1).

**Table 1:** Demographic information

	Control group (*n* = 15)	Test group (*n* = 16)
Age (mean ± SD)	69.2 ± 8.1	67.2 ± 6.3
Male/female	15/0	15/1
Mandible/maxilla	7/8	12/4
Nonmolar/molar	6/9	10/6
Reasons for extraction (Periodontally‐compromised/fracture)	11/4	8/8

### 3.1 Clinical healing

The courses of healing were generally uneventful in all patients. No signs of infection were observed when suture materials were removed. All d‐PTFE membranes were stably maintained in recipient sites until removal. At the time of the membrane removal, the area of the membrane exposed to the oral cavity became larger compared to when ARP procedure had been finished. The membrane surface was covered with a thin layer of yellowish plaque and the margin of mucosal tissue interfacing with the membrane was slightly reddened. All membranes were easily removed using a pincette without local anesthesia. The underlying soft tissue beneath the membrane was generally reddish in color and appeared friable (Figure 1). Bone substitute particles were observable through the thin underlying soft tissue in two patients, but epithelialization was completed without any event.

### 3.2 Radiographic analysis

The data for horizontal and vertical ridge changes are presented in Table 2 and Supporting Information Table S1.

**Table 2:** Horizontal and vertical ridge changes in the control and the test groups (in mm)

	Levels	Control group	Test group	*P* value
Horizontal changes	1 mm	5.4 ± 3.9	2.5 ± 1.9	**.021**
		[3.9, (2.6, 7.8)]	[2.3, (0.6, 4.3)]	
	3 mm	2.4 ± 2.9	1.9 ± 2.0	.572
		[1.4, (1.1, 2.8)]	[1.4, (0.3, 3.0)]	
	5 mm	1.0 ± 0.7	1.4 ± 1.4	.711
		[1.1, (0.6, 1.4)]	[0.8, (0.3, 2.3)]	
Vertical changes	VHB	2.6 ± 2.5	1.1 ± 1.5	.060
		[1.3, (0.8, 4.0)]	[0.5, (0.3, 1.7)]	
	VHM	1.0 ± 1.5	1.0 ± 1.5	.520
		[0.9, (0.5, 1.7)]	[0.5, (0, 1.3)]	
	VHL	1.1 ± 1.5	0.5 ± 1.8	.232
		[1.3, (0.4, 1.9)]	[0.4, (0. 1.2)]	

Data are expressed as mean ± SD [median, (first and third quartiles)].

Bold face indicates statistical difference between the control and the test groups.

At baseline, the median width of the horizontal ridge in the control group was 11.6 mm (Q1 = 9.6; Q3 = 14.6 mm) at HW1, 13.1 mm (Q1 = 11.6; Q3 = 15.8 mm) at HW3, and 14.0 mm (Q1 = 12.4; Q3 = 17.1 mm) at HW5. The corresponding values in the test group were 6.3 mm (Q1 = 4.5; Q3 = 10.1 mm), 11.0 mm (Q1 = 8.1; Q3 = 13.4 mm), and 12.7 mm (Q1 = 8.4; Q3 = 14.8 mm), respectively. After 4 months of healing, the mean width of the horizontal ridge in the control group was 6.3 mm (Q1 = 4.1; Q3 = 8.9 mm) at HW1, 11.0 mm (Q1 = 8.1; Q3 = 12.8 mm) at HW3, and 13.0 mm (Q1 = 11.1; Q3 = 16.1 mm) at HW5. The corresponding values in the test group were 4.2 mm (Q1 = 1.0; Q3 = 8.0 mm), 7.0 mm (Q1 = 6.1; Q3 = 11.4 mm), and 11.1 mm (Q1 = 8.1; Q3 = 13.5 mm), respectively.

**Figure 4:**
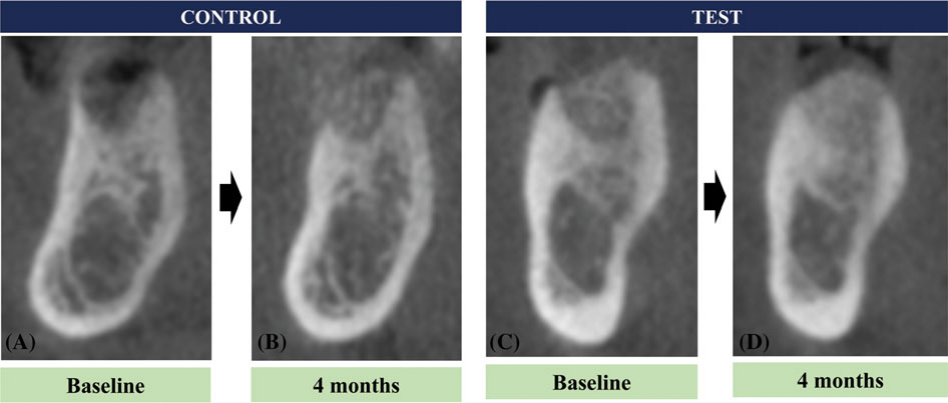
Representative radiographic images in the control group immediately after extraction (A) and after 4 months (B) and in the test group immediately after alveolar ridge preservation (C) and after 4 months (D)

The decrease in the median horizontal ridge width at HW1 was significantly larger in the control group (median = 3.9; Q1 = 2.6; Q3 = 7.8 mm) than in the test group (median = 2.3; Q1 = 0.6; Q3 = 4.3 mm; *P* = .021). There was no statistical difference in the change of the horizontal ridge at HW3 and −5 between the control and the test group (median = 1.4; Q1 = 1.1; Q3 = 2.8 mm vs median = 1.4; Q1 = 0.3; Q3 = 3.0 mm at HW3, median = 1.1; Q1 = 0.6; Q3 = 1.4 vs median = 0.8; Q1 = 0.3; Q3 = 2.3 mm at HW5; *P >* .05 for both comparisons; Figure 4, Table 2).

The median VHB, VHM, and VHL in the control group were 1.3 mm (Q1 = 0.8; Q3 = 4.0 mm), 0.9 mm (Q1 = 0.5; Q3 = 1.7 mm), and 1.3 mm (Q1 = 0.4; Q3 = 1.9 mm), respectively. The corresponding values in the test group were 0.5 mm (Q1 = 0.3; Q3 = 1.7 mm), 0.5 mm (Q1 = 0; Q3 = 1.3 mm), and 0.4 mm (Q1 = 0; Q3 = 1.2 mm), respectively. There were no significant between‐group differences in vertical changes (*P* > .05; Figure 4, Table 2).

#### 3.2.1 Dimensional changes in nonmolar and molar sites

The data for dimensional changes in nonmolar and molar sites are presented in Table 3. Due to a small sample size, descriptive statistics were used. Generally, molar sites underwent greater dimensional changes compared to nonmolar sites in both the test and the control groups, especially at the HW1 level. For nonmolar sites, the median changes at HW1 were 3.1 mm (Q1 = 1.5; Q3 = 5.3 mm) in the control group and 1.5 mm (Q1 = 0.6; Q3 = 2.3 mm) in the test group. For molar sites, these changes were 7.0 mm (Q1 = 3.1; Q3 = 7.8 mm) in the control group and 4.6 mm (Q1 = 4.3; Q3 = 4.6 mm) in the test group.

**Table 3:** Horizontal and vertical ridge changes in nonmolar and molar sockets of the control and the test groups (in mm)

		Control group		Test group	
		Nonmolar (*n* = 6)	Molar (*n* = 9)	Nonmolar (*n* = 10)	Molar (*n* = 6)
Horizontal changes	1 mm	3.9 ± 3.2	6.5 ± 4.2	1.6 ± 1.2	4.1 ± 1.9
		[3.1, (1.5, 5.3)]	[7.0, (3.1, 7.8)]	[1.5, (0.6, 2.3)]	[4.6, (4.3, 4.6)]
	3 mm	1.8 ± 1.1	2.9 ± 3.7	0.8 ± 0.8	3.8 ± 2.0
		[1.6, (1.4, 2.2)]	[1.4, (0.8, 3.2)]	[0.5, (0, 1.5)]	[3.8, (3.0, 4.1)]
	5 mm	1.0 ± 0.9	1.0 ± 0.6	0.6 ± 0.7	2.6 ±1.4
		[1.0, (0.3, 1.6)]	[1.1, (0.6, 1.3)]	[0.3, (0.2, 1.0)]	[2.8, (2.2, 2.9)]
Vertical changes	VHB	1.9 ± 2.4	3.0 ± 2.6	0.3 ± 0.4	2.5 ± 1.6
		[1.2, (0.8, 1.8)]	[2.3, (0.9, 5.7)]	[0.3, (0, 0.4)]	[2.1, (1.7, 2.3)]
	VHM	1.3 ± 1.1	0.9 ± 1.8	0.9 ± 1.4	1.2 ± 1.9
		[1.0, (0.7, 1.4)]	[0.8, (0.4, 1.9)]	[0.5, (0, 1.3)]	[0.5, (0, 1.3)]
	VHL	0.9 ± 0.8	1.3 ± 1.8	−0.3 ± 1.7	1.9 ± 1.0
		[1.0, (0.2, 1.4)]	[1.6, (0.9, 1.9)]	[0.1, (0. 0.3)]	[1.9, (1.2, 2.8)]

Data are expressed as mean ± SD [median, (first and third quartiles)].

### 3.3 Implant‐related outcome

Implants were placed in 13 patients in the control group and 15 patients in the test group. All installed implants did not exhibit rotational or vertical mobility. The details for implant diameter and length are presented in Supporting Information Table S1. Nine control sites and two test sites required bone augmentation at the time of implant placement; this frequency was statistically different between the groups (*P* < .001). In two test sites receiving bone augmentation, one had a dehiscence defect and the other had a fenestration in the apical area of the osteotomy. In the control group, all defects were dehiscence defects.

### 3.4 Bone core biopsy

**Figure 5:**
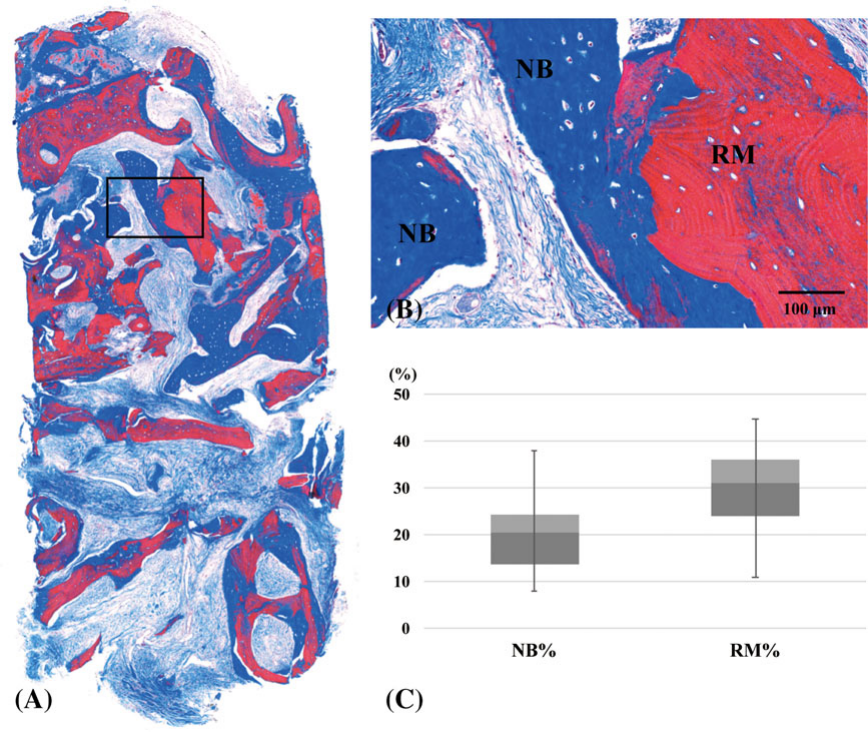
Representative histologic views and histomorphometric analysis in the test group. Images represent the entire specimen from the core biopsy procedure (A), a high magnification view of the boxed area in panel A (B), and a histomorphometric analysis of the core biopsy specimens (C). NB, newly formed bone; RM, residual bone substitute particle; NB%, percentage of newly formed bone; RM%, percentage of residual bone substitute material

Biopsies were taken from nine patients in the test group. Newly formed bone was observed in close contact with residual bone substitute particles. Various amount of marrow tissue formation was observed. Inflammatory cells were rarely observed. Histomorphometric values of NB, RM, and SB were 19.52% ± 9.15%, 21.17% ± 11.20%, and 46.40% ± 15.86%, respectively (Figure 5).

## 4 DISCUSSION

The present study evaluated the effects of ARP with a d‐PTFE membrane and freeze‐dried irradiated allogenic bone substitute material on sockets with bone deficiency, revealing that this ARP method (1) significantly reduced horizontal ridge resorption at the most coronal level, (2) led to a tendency of less ridge resorption in both molar and nonmolar sites, and (3) significantly decreased the need for bone augmentation at the time of implant placement.

Previous studies have demonstrated successful bone regeneration using e‐PTFE membrane, but also reported potentially detrimental effects when it is exposed to the oral environment.^28^ Unfavorable effects are likely derived from the porous structure of e‐PTFE membrane, which facilitates bacterial colonization.^29^ In contrast, the low porosity of d‐PTFE membrane (<0.3 μm) makes it resistant to bacterial infiltration,^17^ which enables the intentional exposure of the membrane in ARP procedures.^30^ Previous studies regarding ARP using d‐PTFE membrane have demonstrated promising results in terms of clinical and radiological ridge dimension as well as histomorphometry^11^, ^14^, ^31^, ^32^; however, this evidence is still limited.^31^

In previous clinical studies, ARP using d‐PTFE membrane demonstrated that the changes of horizontal and vertical ridge dimension following ARP ranged from −0.3 to −3.8 mm and from −1.31 to +0.45, respectively.^11^, ^14^, ^31^, ^32^ This disparity, especially in horizontal width, may be attributable to different surgical techniques, bone substitute materials, and evaluation methodology. Shrinkage in ridge width and height in the present study were in the range of the abovementioned previous studies. It should be noted that previous studies did not include a negative control (eg, a group with naturally healed sockets). In previous systematic reviews with meta‐analyses, the mean differences in horizontal ridge width and the vertical ridge height between the ARP‐received sockets and the naturally healed sockets were 1.31 to 1.89 mm and 0.74 to 2.07 mm, respectively, favoring ARP compared to natural healing.^3^, ^7^, ^23^, ^33^ The present study confirms a statistically significantly decrease in horizontal shrinkage (approximate difference at HW1 between the test and the control group: 2.6 mm), but no significant changes in vertical shrinkage.

Moreover, when nonmolar and molar sites were pooled separately, the test group showed less dimensional change in both tooth sites compared with the control group, especially at HW1 level. Based on these findings, the present ARP seems to be effective in managing nonmolar and molar sockets with bone deficiencies, even though the statistical difference was not analyzed due to a small sample size. Particularly, molar sockets tended to have severe resorption without ARP compared with nonmolar sockets, which indicates that ARP might be a priority treatment option for a molar socket with bone deficiency.

A recent systematic review demonstrated a decreased relative risk for further bone augmentation when ARP was performed,^34^ which is in line with the findings of the present study. Even though the present study evaluated sockets with a substantial bone deficiency, the test group demonstrated statistically less need for bone augmentation at the time of implant placement compared with the control group (13.3% vs 69.2%); this indicates that a socket with bone deficiency may have a high chance for requiring bone augmentation at implant placement if it undergoes natural healing. Considering that bone substitute grafting and membrane placement are relatively less demanding and less time‐consuming procedures at tooth extraction than at implant placement, ARP for the sockets with bone deficiency may lead to less morbidity for the patients and less surgical difficulty for the clinicians. Previously, the presence of erratic extraction sockets was demonstrated,^35^ which also represents the potential benefit of APR for sockets with bone deficiency.

The characteristics of the tissue beneath a placed d‐PTFE membrane are of significant clinical interest. After the removal of the membrane, the underlying tissue appeared reddish in color and friable in texture. One study investigated the histological characteristics of this tissue, revealing that it was composed of a dense connective matrix with a large number of fibroblasts and inflammatory cells, but no epithelial cells.^36^ The role of this tissue has yet to be completely elucidated, but it appears to act like provisional matrix for epithelialization by separating the bone substitute material and the oral environment. In the present study, epithelialization was achieved in all cases without the exposure of the bone substitute particles.

The amount of newly formed bone in the biopsy specimens amounted to 19.52% ± 9.15%, but no comparative analysis was performed due to the lack of biopsy in the control group. Previous studies have reported varying degrees of new bone formation in sockets grafted with freeze‐dried bone allograft, including <20%,^37^ 24.69% ± 15.92%,^11^ 28% ± 14%.^38^ Between‐study variability might be influenced by the type of socket, socket dimension, the amount of bone deficiency in the socket walls, the angle of the core biopsy, and the number of patients.

Flap elevation in the present study may be considered detrimental to ARP due to disruption of the blood supply. Although ARP can be successfully performed without flap elevation in most cases of intact or minimally damaged sockets,^12^ flap elevation may be beneficial for sockets with a substantial amount of bone loss to achieve thorough debridement. Moreover, the systematic review by Avila‐Ortiz and colleagues demonstrated that flap elevation was not detrimental to ARP.^3^

In many studies on ARP in sockets of nonmolar teeth with minimal bone deficiency, resorbable collagen membranes have been used.^26^, ^27^, ^38^, ^39^ However, resorption of a collagen membrane over time may influence the maintenance of ridge dimension, especially for sockets with substantial bone deficiency; instead, nonresorbable d‐PTFE membrane may be more advantageous in this situation. However, it should be also considered that this type of membrane requires an additional intervention, that is, removal of the membrane; however, anesthesia was not required for removal in the present study.

One of the limitations of the present study is a male‐centered demographic (only one female patient). However, a previous study indicated that sex does not influence bone resorption after ARP using a d‐PTFE membrane.^40^ Another limitation is that sockets with bone deficiency are hard to standardize even though the present study followed a previously published classification. Nevertheless, the results of the present study are valuable as they represent patient findings relevant to an everyday clinical setting.

## 5 CONCLUSION

ARP with a d‐PTFE membrane and freeze‐dried irradiated allogenic bone substitute material in sockets with bone deficiency reduced horizontal hard tissue resorption and the need for bone augmentation at the time of implant placement.

## CONFLICT OF INTEREST

No conflict of interest.

## Supporting information


**Supporting Information Table S1** The detailed demographic of the included patients
