# Supplementary material for: Alveolar ridge preservation using an open membrane approach for sockets with bone deficiency: A randomized controlled clinical trial
Source: Clin Implant Dent Relat Res. 2018 Nov 5;21(1):175–82. doi: 10.1111/cid.12668 (PMC6587521; doi:10.1111/cid.12668)
Supplement: Supplementary file 1 — Supporting Information Table S1 The detailed demographic of the included patients [file CID-21-175-s001.docx]

Supplement 1. The detailed demographic of the included patients

| No. | Sex | Age | Sites (tooth no.) | Change at HW1 (mm) | Change at HW3 (mm) | Change at HW5 (mm) | Change at VHB (mm) | Change at VHM (mm) | Change at VHL (mm) | Implant (system/diameter/length) | Defect type at implant placement |
| --- | --- | --- | --- | --- | --- | --- | --- | --- | --- | --- | --- |
| Control group | | | | | | | | | | | |
| 1 | M | 70 | 16 | 14.80 | 11.94 | 1.25 | 0.94 | 4.06 | 3.75 | Osstem TS3 / 5 / 10 | dehiscence |
| 2 | M | 68 | 47 | 7.81 | 3.19 | 2.06 | 2.31 | 1.94 | 1.31 | Osstem TS3 / 5 / 10 | none |
| 3 | M | 42 | 17 | 2.00 | 0.75 | 1.13 | 0.63 | 0.63 | 1.56 | Osstem TS3 / 5 / 10 | none |
| 4 | M | 80 | 45 | 9.81 | 0.31 | 0.00 | 0.31 | -0.25 | 2.81 | Osstem TS3 / 4.5 / 8.5 | dehiscence |
| 5 | M | 77 | 34 | 0.88 | 1.69 | 2.06 | 1.13 | 0.69 | 0.00 | Snucone AF+B / 4.3 / 12 | dehiscence |
| 6 | M | 71 | 46 | 7.69 | 2.50 | 0.50 | 5.69 | 0.94 | 0.00 | Snucone AF+B / 5.3 / 10 | dehiscence |
| 7 | M | 70 | 16 | 2.81 | 0.13 | 0.63 | 6.94 | 0.81 | 1.94 | Dentis OneQ / 5.3 / 10 | none |
| 8 | M | 67 | 27 | 3.88 | 2.38 | -0.06 | 0.00 | 1.19 | 0.00 | Snucone AF+B / 5.3 / 10 | dehiscence |
| 9 | M | 68 | 15 | 5.75 | 0.19 | 0.00 | 1.94 | 0.87 | 0.87 | Osstem TS3 / 4.5 / 11.5 | dehiscence |
| 10 | M | 65 | 45 | 2.33 | 1.42 | 1.00 | 6.67 | 1.42 | 1.42 | None | - |
| 11 | M | 73 | 46 | 3.21 | 1.39 | 1.60 | 2.39 | -2.72 | -2.65 | Osstem TS3 / 5 / 10 | dehiscence |
| 12 | M | 71 | 24 | 9.29 | 3.51 | 1.74 | 0.63 | 0.14 | 1.17 | None | - |
| 13 | M | 62 | 45 | 1.21 | 1.39 | 1.05 | 1.19 | 3.27 | 2.00 | Dentis OneQ / 4.2 /8 | dehiscence |
| 14 | M | 74 | 26 | 3.13 | 1.38 | 1.02 | 1.25 | 0.37 | 0.85 | None | - |
| 15 | M | 71 | 26 | 6.99 | 4.31 | 1.10 | 6.33 | 2.06 | 1.91 | Osstem TS3 / 5 / 10 | dehiscence |
| Test group (dPTFE + freeze-dried allogenic bone) | | | | | | | | | | | |
| 1 | M | 68 | 27 | 0.63 | 0.94 | 0.44 | 1.40 | 0.00 | 1.40 | Osstem TS3 / 5 / 11.5 | none |
| 2 | M | 70 | 14 | 0.81 | 0.13 | 1.25 | -0.10 | 1.30 | 0.30 | Osstem TS3 / 3.5 / 13 | none |
| 3 | M | 67 | 24 | 2.50 | 1.75 | 0.00 | 0.30 | 0.90 | 0.00 | Osstem TS3 / 4 / 10 | none |
| 4 | M | 69 | 33 | 0.19 | 0.00 | 0.00 | 0.10 | -0.80 | 0.70 | Dentis OneQ / 3.9 / 12 | none |
| 5 | F | 67 | 23 | 3.75 | 0.75 | 0.44 | 0.30 | 2.50 | 0.00 | Dentis OneQ / 3.9 / 10 | none |
| 6 | M | 69 | 44 | 0.63 | 0.31 | 0.25 | 0.30 | 0.00 | 0.00 | Dentis OneQ / 3.9 / 12 | fenestration |
| 7 | M | 66 | 15 | 0.31 | 0.00 | 0.19 | 0.00 | 0.00 | 0.20 | Osstem TS3 / 4 / 11.5 | none |
| 8 | M | 71 | 47 | 4.56 | 4.25 | 2.00 | 1.90 | 0.00 | 3.00 | Osstem TS3 / 5 / 10 | none |
| 9 | M | 69 | 25 | 2.19 | 1.56 | 2.13 | 0.50 | 3.80 | -5.00 | Osstem TS3 / 4 / 13 | none |
| 10 | M | 57 | 22 | 2.38 | 2.13 | 1.31 | 1.30 | 0.00 | 0.20 | Osstem TS3 / 3.5 / 11.5 | none |
| 11 | M | 71 | 16 | 4.19 | 3.81 | 2.88 | 2.30 | 4.90 | 2.30 | Snucone AF+B /5.3 / 10 | none |
| 12 | M | 68 | 36 | 4.56 | 7.00 | 4.88 | 5.60 | 1.40 | 0.60 | Osstem TS3 / 5 / 10 | dehiscence |
| 13 | M | 49 | 27 | 6.19 | 2.75 | 2.88 | 2.30 | 0.00 | 1.10 | Dentis OneQ / 5.2 / 10 | none |
| 14 | M | 69 | 24 | 2.19 | 0.00 | 0.38 | 0.40 | 1.20 | 0.00 | Osstem TS3 / 4 / 11.5 | none |
| 15 | M | 69 | 25 | 0.63 | 1.31 | 0.31 | -0.30 | 0.00 | 0.50 | Osstem TS3 / 4.5 / 10 | none |
| 16 | M | 73 | 16 | 4.63 | 3.75 | 2.81 | 1.60 | 0.90 | 2.90 | Dentis OneQ / 5.2 / 10 | none |

HW1, 3 and 5: horizontal ridge width at a level of 1, 3, and 5 mm below the ridge crest, assessed using CBCT

VHB, VHM, and VHL: vertical ridge height at the buccal, mid, and lingual crests, assessed using CBCT
